# The TOC GTPase Receptors: Regulators of the Fidelity, Specificity and Substrate Profiles of the General Protein Import Machinery of Chloroplasts

**DOI:** 10.1007/s10930-019-09846-3

**Published:** 2019-06-14

**Authors:** Danny J. Schnell

**Affiliations:** Department of Plant Biology, Michigan State University, East Lansing, MI USA

**Keywords:** Chloroplast biogenesis, Chloroplast protein import, Protein targeting, Protein quality control, Transit peptide, Endosymbiosis

## Abstract

More than 2500 nuclear encoded preproteins are required for the function of chloroplasts in terrestrial plants. These preproteins are imported into chloroplasts via the concerted action of two multi-subunit translocons of the outer (TOC) and inner (TIC) membranes of the chloroplast envelope. This general import machinery functions to recognize and import proteins with high fidelity and efficiency to ensure that organelle biogenesis is properly coordinated with developmental and physiological events. Two components of the TOC machinery, Toc34 and Toc159, act as the primary receptors for preproteins at the chloroplast surface. They interact with the intrinsic targeting signals (transit peptides) of preproteins to mediate the selectivity of targeting, and they contribute to the quality control of import by constituting a GTP-dependent checkpoint in the import reaction. The TOC receptor family has expanded to regulate the import of distinct classes of preproteins that are required for remodeling of organelle proteomes during plastid-type transitions that accompany developmental changes. As such, the TOC receptors function as central regulators of the fidelity, specificity and selectivity of the general import machinery, thereby contributing to the integration of protein import with plastid biogenesis.

## Introduction

**Fig. 1:**
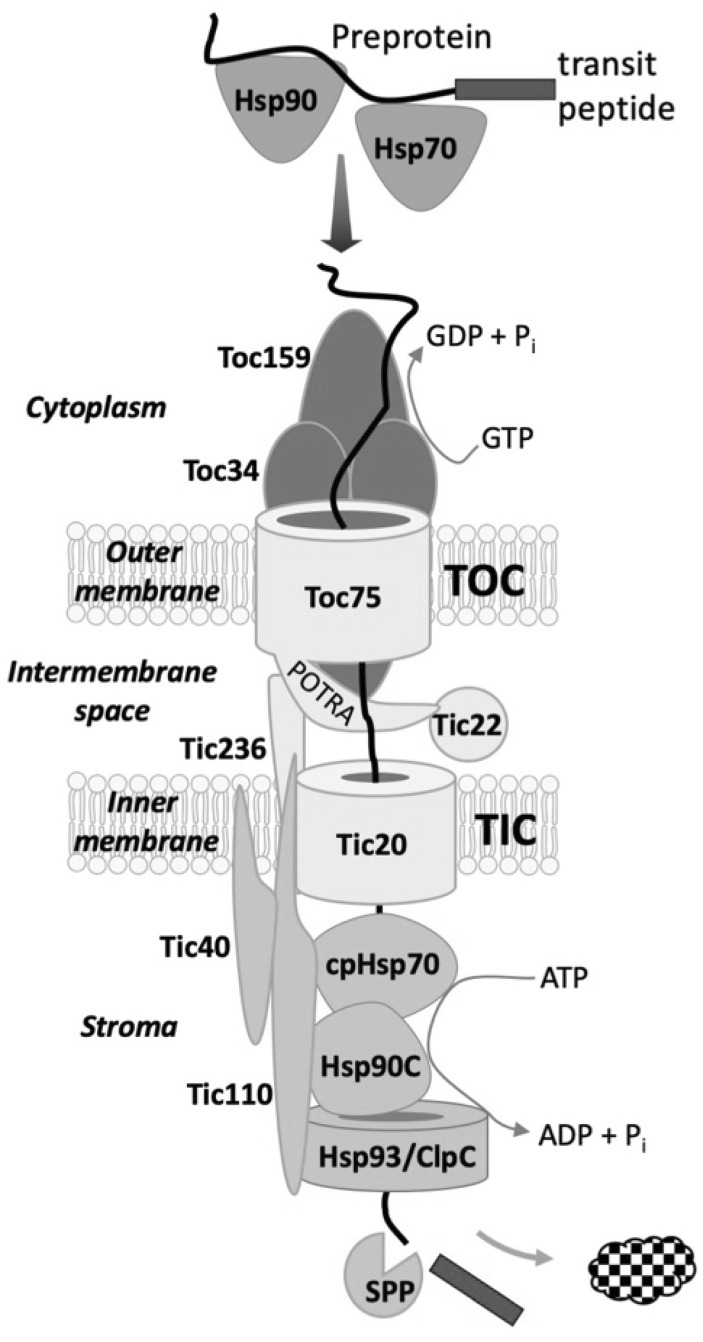
The core, conserved components of the TOC-TIC general import machinery of chloroplasts. Nuclear encoded preproteins are targeted to chloroplasts with the assistance of cytosolic complexes containing Hsp70 and Hsp90 chaperones. The Toc34 and Toc159 receptors initiate import by recognizing the transit peptide of the preprotein, and their GTPase activities control the commitment of the preprotein to membrane translocation via the Toc75 and Tic20 channels in the outer and inner membranes, respectively. Translocation occurs simultaneously through TOC-TIC supercomplexes formed by Tic236, which spans the intermembrane space and binds to both TOC and TIC complexes. The Toc75 POTRA domains and the small chaperone, Tic22 facilitate passage of the preprotein through the intermembrane space. The TIC complex also contains Tic110 and Tic40, which assemble the ATP-dependent import motor in the stroma. The import motor consists of the cpHsp70, Hsp93/ClpC, and Hsp90C chaperones. The transit peptide is cleaved by the stromal processing peptidase (SPP) once it enters the stroma

The endosymbiotic evolution of chloroplasts from a cyanobacterial ancestor resulted in the transfer of the majority of genes from the bacterial endosymbiont to the host cell nucleus. As a consequence, plants evolved at least four distinct pathways to mediate the targeting of thousands of nuclear encoded proteins back to the organelle [1, 2]. The vast majority of proteins are targeted via the interaction of their intrinsic targeting signals (transit peptides) with the general import machinery of chloroplasts, which consists of translocon complexes in the outer (TOC) and inner (TIC) envelope membranes (Fig. 1) [1, 3–9]. TOC and TIC are physically linked to form supercomplexes that mediate direct transport of preproteins from the cytoplasm into the organelle interior [10–13]. Chaperone complexes in the cytoplasm, intermembrane space, and stroma interact with TOC-TIC to facilitate import and avoid misfolding or mistargeting of preproteins as they traverse the outer and inner envelope [8, 14]. The TOC-TIC general import machinery also interfaces with six known suborganellar targeting pathways that function to route proteins to the thylakoid or inner envelope membranes subsequent to import [2]. As such, TOC-TIC serves as a central hub for coordinating the import and suborganellar targeting of proteins required for all aspects of plastid biogenesis.

The central role of TOC-TIC in organelle biogenesis dictates that import operate with high fidelity, while accommodating highly variable import fluxes and a wide range of import substrates. The core components of the TOC-TIC machinery, including the Toc75 and Tic20 protein conducting channels in the outer and inner envelope membranes, respectively (Fig. 1), appear to have been adapted from bacterial protein export systems [13, 15–18]. The specificity and fidelity of import was bestowed by the addition of the Toc159 and Toc34 receptors that assemble with Toc75 to mediate the initial recognition of transit peptides at the chloroplast surface and control access to the membrane translocation machinery through their intrinsic GTPase activities (Fig. 1) [19]. The TOC receptor family has expanded as the chloroplast diversified into a larger family of plastid types to generate import complexes with specificities for distinct classes of proteins [6]. This review will focus on the latest developments in our understanding of the mechanism, diversity and regulation of the Toc34 and Toc159 transit peptide receptors and their critical role in balancing the profiles of imported proteins that are required for plastid-type transitions as the organelle proteome is remodeled in response to physiological or developmental changes.

## The General Import Pathway

A general overview of the core, conserved components of the general import machinery is presented in Fig. 1. Chloroplast preproteins are imported post-translationally subsequent to synthesis on cytosolic ribosomes, and their transit to the chloroplast surface is facilitated by complexes containing members of the Hsp70 and Hsp90 families of molecular chaperones [14, 20–23]. The initial interaction of preproteins at the chloroplast surface is mediated by Toc34 and Toc159 [3, 8]. Toc34 and Toc159 are members of the translation factor (TRAFAC)-related superclass, which includes many major regulatory GTPases [24]. Both receptors are anchored in the outer membrane with cytosolically exposed GTPase domains, which contain transit peptide binding sites [25–30]. The two receptors assemble with the Toc75 channel to form large ~ 800 kDa complexes in the outer membrane, with Toc34 and Toc75 in stoichiometric excess to Toc159 [10–12]. Studies using synthetic transit peptides and transit peptide mutations indicate that Toc159 and Toc34 have a higher affinity for the N-terminal and C-terminal regions of a model transit peptide, respectively, demonstrating that the two receptors can bind the transit peptide simultaneously during preprotein recognition in the cytosol [31, 32].

The initial interaction with the receptors leads to partial insertion of the transit peptide across the outer membrane, with the mid-region of the transit peptide in contact with Toc75 and the N-terminal region of the transit peptide in contact with the Tic20 channel at the inner membrane [12, 33]. ATP hydrolysis promotes the association of the preprotein with the import associated chaperone complex in the stroma, and the chaperones function as a motor to drive import across the envelope [34–39]. The import motor is tethered to the TIC complex by the Tic110 scaffold protein and the Tic40 co-chaperone [40–49]. The simultaneous translocation of preproteins through TOC and TIC is facilitated by Tic236, a protein that spans the intermembrane space and associates with both translocons to form supercomplexes at membrane contact sites [13, 18].

Once exposed to the stroma, the transit peptide is processed by the stromal processing peptidase, and the polypeptide folds and assembles with other polypeptides in the stroma or engages additional suborganellar targeting pathways to the thylakoid or inner envelope membranes [1, 2]. A number of additional proteins that interact with the core machinery to facilitate import or suborganellar targeting are described in detail in other recent reviews [1, 3–9]. These components are not included here because they play accessory roles or are present only in select species and are therefore not considered core components of the general import machinery.

## The TOC GTPases as Regulators of Import Fidelity

The role of the TOC receptor GTPase activity was first revealed by the observation that non-hydrolysable analogues inhibit protein import [50–52]. Site-specific cross-linking in the presence of GTPγS demonstrates that the transit peptide partially inserts across the TOC-TIC machinery, engaging both the Toc75 and Tic20 channels at the outer and inner membranes, respectively [12]. Therefore, GTP hydrolysis does not provide the energy for the initial insertion of the preprotein into the protein conducting channels. However, subsequent ATP-dependent import is blocked by GTPγS, suggesting that GTP hydrolysis at the TOC receptors acts as a checkpoint prior to the commitment of the preprotein to import into the organelle [12]. Consistent with this conclusion, preprotein binding in the presence of GTPγS is reversible and precludes the high affinity, irreversible binding to the import motor chaperones observed in the presence of ATP [50, 51]. As such, the receptors function as GTP-regulated switches that regulate the check-point for commitment to import.

**Fig. 2:**
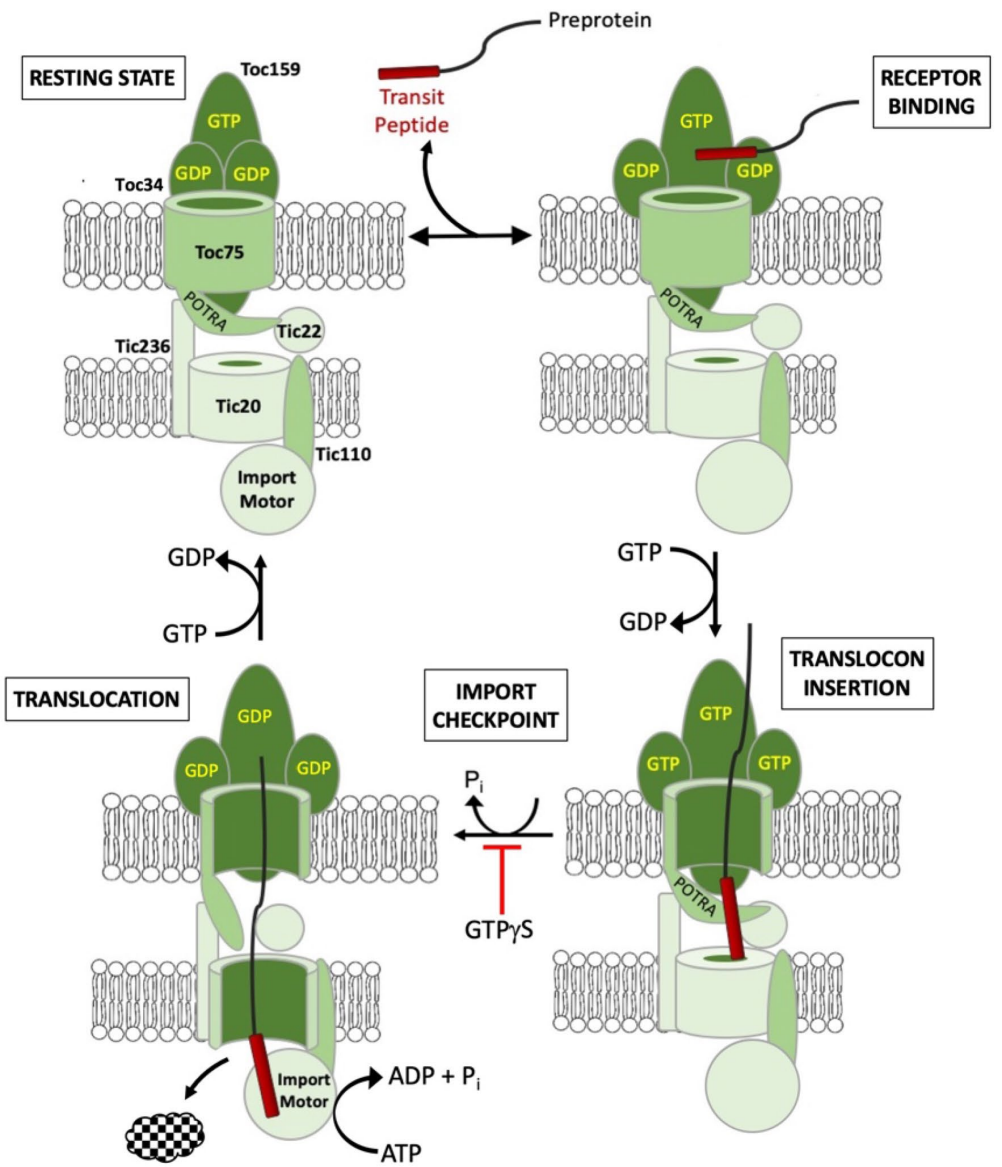
A model for the role of the TOC GTPase receptors as regulators of to the general import machinery. The resting state of the TOC complex contains the Toc159_GTP_ and Toc34_GDP_ preprotein receptors (Resting State). The receptors bind to preproteins simultaneously via interactions of the transit peptide with the GTPase domains of the receptors (Receptor Binding). Transit peptide binding triggers the dissociation of Toc34_GDP_ homodimers, thereby promoting GDP for GTP exchange and allowing the formation of Toc34 _GTP_ -Toc159 _GTP_ heterodimers. This conformational change allows the preprotein to insert into the translocons via the Toc75 and Tic20 channels (Translocon Insertion). GTP hydrolysis at the receptors is required for the preprotein to engage the import motor in the stroma, thereby serving as a fidelity checkpoint in the import reaction (Import Checkpoint). The chaperone components of the import motor drive unidirectional import of the preprotein into the stroma via cycles of ATP hydrolysis (Translocation). Once import is complete, the TOC-TIC machinery resets to the resting state for another round of import

A picture of the mechanism of the TOC GTPase switch has now emerged from detailed biochemical and structural studies of Toc159 and Toc34 (Fig. 2). Toc34 forms homodimers via interactions between GTPase domains both in vitro and in vivo [53–55]. The GTP/GDP binding site on the receptor lies at the dimer interface, precluding nucleotide exchange. The GDP-bound homodimer is proposed to represent the resting state of the receptor in the absence of transit peptide binding (Fig. 2, Resting State) because the relative affinity of Toc34 for GDP is higher than for GTP, and available crystal structures capture GDP caged at the dimer interface [32, 54]. Binding of the preprotein transit peptide to Toc34 promotes dissociation of Toc34 homodimers and stimulates GDP/GTP exchange and GTP hydrolysis (Fig. 2, Receptor Binding) [56]. However, the transit peptide does not appear to function as either a classic GTPase exchange factor or activator protein. Rather, it appears that dissociation of the Toc34 dimers is sufficient to open the nucleotide binding sites and promote GDP for GTP exchange driven by the relatively high ratio of GTP to GDP concentrations in the cytoplasm. Although dimers of the Toc159 GTPase domain have been identified in vitro [57], GTP/GDP binding studies suggest that it exists primarily as a GTP-bound monomer in the absence of transit peptide binding [32]. Toc159 does form heterodimers with Toc34 in vitro. However, Toc34 is proposed to favor homodimerization over heterodimerization in the absence of transit peptide binding in TOC complexes based on binding affinity measurements [32].

These observations have led to a revised model of preprotein recognition by the TOC translocon (Fig. 2). In this model, the TOC receptors are proposed to play two key roles in monitoring the fidelity of import. First, the receptors control access of the transit peptide to the TOC-TIC channels. The resting state of the translocon corresponds to Toc34_GDP_ homodimers and Toc159_GTP_ monomers (Fig. 2, Resting State). Simultaneous binding of distinct regions of the transit peptide to the receptors triggers dissociation of Toc34_GDP_ homodimers and promotes nucleotide exchange to Toc34_GTP_ (Fig. 2, Receptor Binding) [25, 28, 58, 59]. In the model, this conformational arrangement of GTP-bound receptors allows the transit peptide to insert into the translocons and engage the Toc75 and Tic20 channels, consistent with the results from site-specific crosslinking (Fig. 2, Translocon Insertion) [12]. This stage also could involve Toc34_GTP_-Toc159_GTP_ heterodimerization, although there is limited information on whether heterodimerization is required as part of the recognition cycle [55, 60, 61]. It should be noted that the initial recognition of the transit peptide by the receptors in the model is transient and functions to provide access for the transit peptide to the translocation channels within TOC-TIC supercomplexes. The transient binding of the transit peptide to the receptors is consistent with the 70–150 μM range of K_D_’s measured for binding of transit peptides by both TOC receptors [32, 62]. As such, Toc34 and Toc159 are envisioned to function more like transit peptide-regulated molecular switches than classic ligand receptors.

GTP hydrolysis appears to play the second key role for the receptors in controlling the initial stages of import. Preprotein binding and crosslinking studies demonstrate that GTP hydrolysis is necessary to allow the transition from the transit peptide interaction with Toc75 and Tic20 to preprotein engagement of the ATP-dependent import motor [12]. Thus, GTP hydrolysis at the receptors appears to induce a conformational change in the translocons that releases the preprotein for import (Fig. 2, Translocation). Although the nature of this conformational change is unknown, one possibility is that GTP hydrolysis regulates interactions of the translocon with the transit peptide in the intermembrane space. On this basis, it has been proposed that the topology of the preprotein during translocon insertion corresponds to binding of the transit peptide to the polypeptide transport associated (POTRA) domains of the Toc75 channel in the intermembrane space [16, 63]. The large membrane-domain of Toc159 also localizes to the intermembrane space and has been shown to be in close proximity to the transit peptide at this stage in import [26]. It is therefore possible that a GTPase-regulated conformational change in Toc159 controls binding to the Toc75 POTRA domains and the transition from translocon insertion to translocation (Fig. 2).

## The Selectivity of TOC-TIC is Determined by TOC Receptor Isoforms

As central regulators of protein import, the TOC GTPases also control the profiles and levels of preprotein import during various stages of plastid development. The Toc34 and Toc159 receptors consist of small gene families, and they assemble with the Toc75 channel to generate translocons with distinct specificities for different classes of preproteins [64–69]. The translocon specificities correlate with classes of preproteins whose import is differentially regulated during chloroplast development [70]. The selectivity of TOC complexes appears to be primarily determined by the Toc159 receptor family [66, 68, 69]. For example, the atToc159 and atToc132 receptors of the Toc159 family in Arabidopsis are required for biogenesis of the photosynthesis machinery and plastid housekeeping functions, respectively [68, 69]. Null mutations in genes encoding different atToc159 and atToc132 family members have distinct impacts on plastid development in various tissues, and deletion or swapping of the cytoplasmic N-terminal acidic domains of the isoforms largely dictates the selectivity of the translocons [64–69]. The acidic domain of Toc159 receptors also is phosphorylated by an outer envelope kinase, KOC1, and *koc1* mutants are impaired in protein import, providing compelling evidence that the TOC receptors are also regulated by phosphorylation [71, 72].

**Fig. 3:**
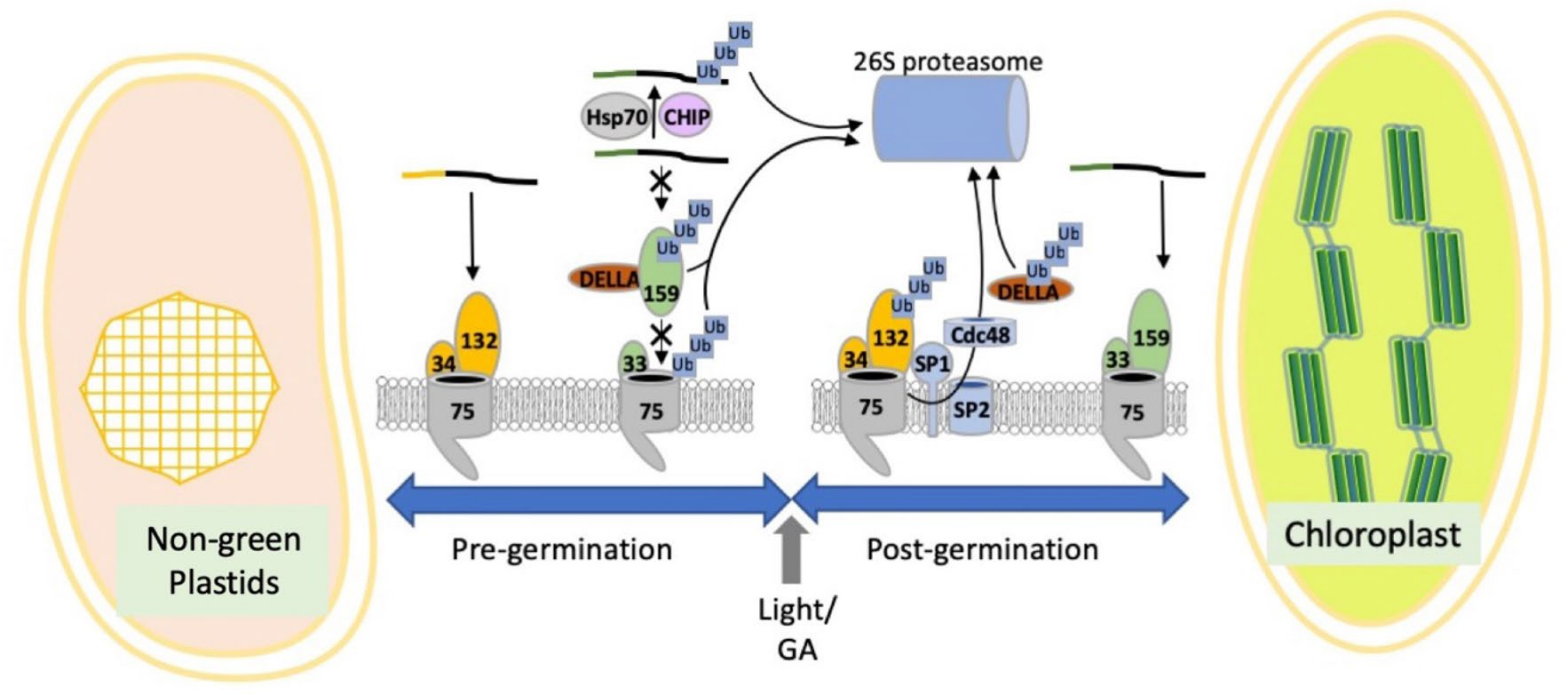
Regulation of the plastid general import machinery by the ubiquitin–proteasome system. The specificity of the import complexes is determined by the Toc34 and Toc159 family of transit peptide receptors. The relative abundance of distinct TOC complexes controls the levels of import of specific classes of preproteins that are required for plastid-type transitions during development. The example provided is for the transition from non-green plastids (e.g. etioplasts or proplastids) to chloroplasts during seed germination and seedling development. Prior to germination (Pre-germination), low levels of gibberellic acid (GA) lead to the accumulation of the DELLA regulators in the cytoplasm. DELLAs prevent the assembly of Toc159-Toc33 complexes (green) involved in the import and assembly of the photosynthetic apparatus by targeting the Toc159 receptor (159) for degradation by the 26S proteasome. Any photosynthetic preproteins that accumulate in the cytoplasm also are targeted for UPS degradation via the combined activities of Hsp70-4 (Hsp70) and the CHIP cytosolic E3 ubiquitin ligase (CHIP). Together, these systems suppress chloroplast development in the dark to prevent premature greening and photooxidation caused by accumulation of chloroplast precursors. Toc132(132) -Toc33(33) complexes required for maintaining basic plastid function (orange) are not regulated by the GA/DELLA system. Increased GA and light trigger germination and seedling greening (Post-germination) and cause UPS degradation of DELLAs. This allows the assembly of Toc159-Toc33 translocons and the import of preproteins required for photosynthesis. Another UPS pathway, involving a RING-type E3 ubiquitin ligase (SP1), the SP2 β-barrel channel, and the cytosolic Cdc48 AAA + ATPase target the Toc132-Toc33 complexes for UPS degradation, thereby balancing the flux of import between preproteins required to build the photosynthetic apparatus and those necessary to maintain basic plastid function during chloroplast biogenesis

The relative abundance of translocon isoforms also appears to be critical in balancing the import of distinct classes of preproteins that are required for proteome remodeling during plastid-type transitions or organelle responses to physiological changes (Fig. 3, Post-germination). The levels of distinct translocons are monitored by a branch of the ubiquitin proteasome system (UPS) that resides in the outer envelope membrane and associates with TOC complexes [73, 74]. At least two UPS associated monitoring systems of TOC complexes have been identified. The first system was discovered in leaf tissues and appears to be critical during early chloroplast development in seedlings. It is composed of suppressor of *ppi1* (SP1), an integral membrane E3 ubiquitin ligase, SP2, a β-barrel membrane protein that is proposed to provide the retrotranslocation channel, and the Cdc48 AAA^+^ ATPase (Fig. 3, Post-germination) [73, 74]. Together, they constitute a chloroplast-associated degradation (CHLORAD) system for selective ubiquitination, extraction, and delivery of TOC complexes to the 26S proteasome for degradation in a manner analogous to the ER-associated degradation (ERAD) system.

A second UPS-associated pathway operates during seed germination (Fig. 3, Pre-germination) [75]. The assembly of the photosynthetic apparatus as plants transition from chemoautotrophic to photoautotrophic growth must be tightly coordinated to avoid photooxidative damage by imbalances in the accumulation of photosensitive components in the organelle. This coordination appears to be controlled by the phytohormone, gibberellic acid (GA). The levels of GA are low in seeds leading to the accumulation of the DELLA family of regulator proteins. DELLAs bind to Toc159 in the cytoplasm and target the receptor for UPS degradation, thereby preventing the premature assembly of protein import complexes that are necessary for the import of photosynthetic proteins. The import of housekeeping proteins, mediated by other TOC isoforms (e.g. containing Toc132), is not regulated by the GA/DELLA system. During germination, GA levels rise, leading to the ubiquitination and degradation of DELLA proteins, thereby allowing Toc159 to assemble and form the functional import complexes that are necessary for chloroplast biogenesis [75].

A distinct UPS pathway also monitors the premature accumulation of photosynthetic preproteins and prevents them from accumulating to toxic levels in the cytoplasm (Fig. 3, Pre-germination) [76]. A similar system also exists in leaf tissues, and likely all other plant tissues. This system consists of the Hsp70 isoform, Hsc70-4, and the CHIP cytosolic E3 ubiquitin ligase. Hsc70-4 selectively recognizes cytosolic preproteins via a motif within the transit peptides, and down-regulation of Hsc70-4 or the inhibition of the 26S proteasome in Arabidopsis results in the accumulation of chloroplast preproteins in the cytosol [76].

## Conclusion

The emerging picture of TOC receptor function and regulation reveals their central role in mediating both the selectivity and fidelity of the general import machinery. Although many details of the molecular mechanism of TOC-TIC function remain to be investigated, it is clear that the evolution and adaptation of the TOC represented a major step in transforming an ancestral bacterial protein export system into an import system to support the new endosymbiont. The role of the receptors in constituting a GTPase import checkpoint in combination with a UPS monitoring system in the cytoplasmic provides a high-fidelity quality control system that avoids preprotein mistargeting and aggregation. This mechanism of controlling the committed step in import represents another remarkable example of a cellular GTPase switch that adapted to respond to recognition of a unique organellar targeting system. The discovery that the TOC receptors also are major targets for regulating import during developmental and physiological changes has revealed an essential role for import in balancing both the levels and types of proteins that are targeted to the organelle. To date, protein import has been studied almost exclusively in chloroplasts, and it is clear that a full picture of both the mechanism and regulation of import will require studies in the more than a dozen other plastid types that are essential for plant growth and development.

## Abbreviations

TOC: Translocon at the outer chloroplast envelope
TIC: Translocon at the inner chloroplast envelope
Hsp: Heat shock protein
POTRA: Polypeptide transport associated
SP: Suppressor of ppi1
UPS: Ubiquitin proteasome system
Cdc48: Cell cycle division 48
ERAD: ER-associated degradation
GA: Gibberellic acid
KOC: Kinase of the outer chloroplast membrane
*Ppi*: Plastid protein import mutant
CHIP: Carboxyl-terminus of Hsp70-interacting protein
